# Relationship between Low Birth Weight Neonate and Maternal Serum Zinc Concentration

**Published:** 2012-04-01

**Authors:** N Khadem, A Mohammadzadeh, A S Farhat, L Valaee, M Khajedaluee, S M R Parizadeh

**Affiliations:** 1 Department of Obstetrics and Gynecology, Neonatal Research Center, Mashhad University of Medical Sciences, Mashhad, Iran; 2 Department of Neonatology, Neonatal Research Center, Mashhad University of Medical Sciences, Mashhad, Iran; 3 General physician, Neonatal Research Center, Mashhad University of Medical Sciences, Mashhad, Iran; 4 Department of Community Health, Mashhad University of Medical Sciences, Mashhad, Iran; 5 Department of Biochemistry and Nutrition, Mashhad University of Medical Sciences, Mashhad, Iran

**Keywords:** Zinc, Low birth weight, Pregnant

## Abstract

**Background:**

Zinc deficiency can lead to clinically relevant disturbances in tissue functions and particularly important for birth weight of neonates. The aim of this study was to determine the relationship between serum zinc in pregnant women and the incidence of low birth weight (<2500 g) in their newborns.

**Methods:**

This case-control study was done on women who delivered low birth weight infants (Cases), and normal birth weight infants (Controls). Blood samples got in all women within 5 minutes of delivery, and assessed the concentration of zinc using electrothermal atomic absorption spectrometry. Serum concentration of zinc was compared.

**Results:**

One hundred and seventeen mothers were enrolled, of them, 65 cases were low birth weight infants (1845±472 g) and 52 were controls (3166± 435 g). Mothers in the case and control groups did not differ in age, body mass index, and socioeconomic or demographic factors. Maternal zinc concentration differed between cases and controls; 753.34±15 µg/l vs. 654.76±12 µg/l respectively. Maternal zinc differed between premature and full term deliveries.

**Conclusion:**

Maternal zinc concentration was shown to affect birth weight and prematurity.

## Introduction

Low birth weight (LBW) is a crucial and a substantial factor contributing to infant mortality.[1][2] Infants with LBW are at increased risks for long term disability and various physical morbidities.[3] Poor nutrition during pregnancy is recognized as an important cause for LBW particularly in developing countries.[4] Deficiencies in micronutrients such as zinc, iron, folic acid and iodine during pregnancy can cause LBW.[5]

Pregnant women in developing countries were shown to consume diets with a lower density of minerals and vitamins. Suboptimal zinc intake may be relatively common throughout the world.[6] Low zinc intake may be especially detrimental during pregnancy due to the role that zinc plays in growth and development. Potential adverse consequences of zinc deficiency during pregnancy include increased maternal mortality, LBW, prolonged labor, spontaneous abortion and prematurity.[6][7] Impaired zinc status during pregnancy was recently found to adversely influence late fetal development.[8]

Evidences suggests that zinc deficiency is one of the important problems within the developed and developing countries.[9] In 1991, a report emphasized the importance of zinc and also indicated that in countries such as Iran, Egypt, Turkey, China, Yugoslavia, and Canada, due to low consumption of red meat, and high consumption of fiber, zinc deficiency was seen quite often.[9] Zinc deficiency in human was reported for the first time in Iran in 1961[10] and in Egypt in 1963.[11]

Pregnant women are facing zinc deficiency more than the other groups, due to having fetus which need zinc for its poor growth.[12] A separate study in 2001 indicated that using supplementary zinc materials by pregnant mother, increased newborn birth weight, and decreased the mortality rate.[12] The aim of this prospective case-controlled study was to determine the relationship between maternal zinc concentrations and neonatal birth weight in a group of mothers having LBW deliveries as compared to a group who delivered normal birth weight infants. If zinc was found to be involved in the pathogenesis of LBW, then supplementation of zinc during pregnancy could possibly be considered to ameliorate this serious morbidity.

## Materials and Methods

We conducted this prospective case-control study at the maternity ward of Imam Reza Hospital and the Neonatal Research Center of Mashhad University for Medical Sciences in Iran. The study protocol was approved by the research office at the university, and written informed consent was obtained from each subject before enrollment. The study was done between May 2006 and February 2007. Mother-infants pairs were included if (i) maternal age was 17-35 years at the time of delivery, and (ii) She had uncomplicated singleton pregnancy.

“Cases” were considered if the birth weight of the newborn was <2500 g without any known underlying risk factors that could explain the reason for the LBW. A “Control” subject was enrolled when a subsequent mother delivered an infant with birth weight =2500 g.

Mothers were not considered for the study if any of the following exclusion criteria were present: A previous history of LBW delivery, twin and multiple pregnancies, preeclampsia and eclampsia, uterine cervical abnormalities, antenatal bleeding, oligo- and polyhydramnios, abnormal maternal conditions prior pregnancies; such as lupus, chronic hypertension, diabetes mellitus, seizure disorders, malignancies, and drug or alcohol abuse.

The investigator did a thorough physical examination of every infant recruited into the study. The infant's weight was taken to the nearest 10 g and the length was recorded to the nearest 0.1 cm. The gestational age was assessed clinically using the Dubowitz method.[13] Medical and social data on mothers were obtained from available information in their records and by direct interviews.

For zinc assays in the first, 5 minutes after delivery, immediately after collection of blood, it was centrifuged at 3000 rpm for 15 min. Plasma was separated and kept frozen at -20°C. The samples were analyzed after all of them collected. Serum zinc concentration was determined by flame atomic absorption spectroscopy (AAS) in a Perkin-Elmer 1100B apparatus (Atomic Absorption Spectrophotometer). All samples were analyzed in duplicate. Our laboratory reference range for serum zinc was ≥500 µg/L, which is similar to that of other laboratories.[14][15][16]

All specimens were de-identified before processing, therefore, laboratory staff was masked to clinical conditions. The data were analyzed using the SPSS software (Version 11.5, Chicago, IL, USA). For quantitative data with normal distribution t-test and with abnormal distribution, Kruskal-Wallis test were used. For qualitative data, X2 test was used.

Multiple linear regression analysis was performed to control potential confounding variables. Statistical significance was considered when p was less than 0.05.

## Results

The study included 117 mother-infant pairs, of them, 65 were cases and 52 were controls. The demographic and clinical characteristics of the study population are listed in Table 1. Maternal zinc concentration (µg/l) differed between cases and controls; 753.34±15 µg/l vs. 654.76±12 µg/l respectively. It was related to either gestational age or birth weight (Table 2).

**Table 1:** Demographic and clinical characteristic of the study population (n=117).

**Variable **	**Case** **(n=65)**	**Control** **(n=52)**	***Pvalue***
A. Maternal			
Age (year)	24±4	25.7±5.4	0.059
BMI (mean±SD)	23.4±3.4	22.9±3.2	0.448
Education (%)			
Illiterate	4	11	0.336
Primary	47	53	
Intermediate	43	32	
University	6	4	
Income (%)			
Monthly income <150$	21	34	0.029
Monthly income<150-500$	69	66	
Monthly income >500$	10	0	
B. Infant			
Gender			
Female (%)	43	53	0.281
Male (%)	57	47	
Birth Weight (g)	1845±472	3167±435	<0.001
Apgar (mean±SD)			
1 minute	7.56±1.33	8.38±0.63	<0.001
5 minute	8.19±1.31	8.94±0.41	<0.001
Gestational Age (wk)	33.4±2.9	39.3±1.4	<0.001

**Table 2:** Maternal serum zinc concentration according to gestational age and birth weight in case and control groups.

**Characteristics**	**Number**	**Zinc concentration (μg/l)**	***P *value**
**Gestational Age (Mean±SD)**			
<32 week	21	783.28±14	
32-37 week	44	769.25±15	<0.001
>37 week	52	654.76±12	
**Birth weight (Mean±SD)**			
<2000 gram	36	790.25±14	
2000-2500 gram	29	753.34±15	<0.001
>2500 gram	52	654.76±12	

On regression analysis, zinc concentrations did not differ between cases and controls (p>0.05) after we controlled for various confounders such as maternal age, body mass index (BMI), educational level, income, neonatal gender, birth weight, gestational age, and Apgar scores.

## Discussion

This study demonstrated zinc concentrations in the serum of mothers who delivered LBW infants differ from mothers who delivered infants with a normal birth weight, and serum zinc level in case group was significantly higher than mothers with normal birth weight and mature infants (p<0.01).

Our results showed that zinc level decreased with increasing gestational period. The maximum decrease in zinc level was observed during weeks of 35-36 of gestational age. These results are similar to other studies.[17][18][19][20] Serum zinc was within normal range in all enrolled women. This could be related to the recent mandate by the Ministry of Health of Iran to provide multivitamins and minerals supplementations to all expectant women.

A study carried out by Diet Health and Knowledge Survey of Iran Ministry of Health in 2001 about micronutrient consumption among the pregnant women in eleven regions of Iran was representative of all geographical areas. Analysis showed that prevalence of maternal zinc deficiency in Mashhad was 43.3% and among others areas of Iran, it was a third grade maternal zinc deficiency. Although significant correlation was found between the age of pregnancy and maternal zinc concentration, so during the third trimester, 44% of pregnant women were zinc deficient.[21]

A total of 12 randomized, controlled intervention trials of supplemental zinc and pregnancy outcome have been reported.[22] Of the 12 trials, 6 found no effect of the zinc intervention on pregnancy outcome. Two studies reported that supplemental zinc significantly improved fetal growth.[23][24] Both studies were done in populations in whom maternal zinc depletion was likely. In the study conducted in India, the birth weight of neonates from women in the placebo group averaged only 2.6 Kg. Neonates of zinc-supplemented mothers were 0.3–0.8 Kg more, depending on the length of time supplemental zinc was provided.[23] If the zinc supplement was initiated in the first trimester, the effect on birth weight was greater if it was initiated in the third trimester.

Although plasma zinc concentration is commonly used as an indicator of zinc status, interpretation of the measurement is difficult because plasma zinc concentration decreased with infection, vigorous exercise, and food intake. Assessment is more difficult during pregnancy because plasma zinc concentration declines in proportion to the increase in plasma volume.[25]

It can be concluded that, when mothers receive zinc supplementation during pregnancy, zinc concentration in the blood plays a role in determining the birth weight of term and preterm infants. Our study determined if such conclusion can apply to zinc depleted mothers.

It is accomplished that an early and progressive decline in serum zinc occurs during gestation and a poor maternal zinc status may limit the metabolic adaptation capacity of women especially during pregnancy. Some previous studies demonstrated that zinc deficiency during pregnancy in early weeks can produce abortion and congenital malformation.[26][27] If this deficiency continues during pregnancy, the growth of the fetus may cause complications at birth.[20] Birth weight depends on many factors such as gender of the neonate, maternal age, race, BMI, maternal weight gain during gestation, smoking, and alcohol consumption. Multiple regression analyses should be performed to control for all of these variables before blaming zinc deficiency for the presence of LBW. Maternal zinc concentration was shown to affect birth weight and prematurity.
